# The dimorphic fungus *Talaromyces marneffei*: An opportunistic killer in Southeast Asia

**DOI:** 10.1371/journal.ppat.1013444

**Published:** 2025-09-10

**Authors:** Lottie Brown, Alex Andrianopoulos, Patrick C. Y. Woo, Thuy Le

**Affiliations:** 1 Institute of Infection and Immunity, City St George’s University of London, School of Health & Medical Sciences, London, United Kingdom; 2 Clinical Infection Unit, St George’s Hospital, St George’s University Hospitals NHS Foundation Trust, London, United Kingdom; 3 School of Biosciences, University of Melbourne, Melbourne, Victoria, Australia; 4 Doctoral Program in Translational Medicine and Department of Life Sciences, National Chung Hsing University, Taichung, Taiwan; 5 The iEGG and Animal Biotechnology Research Center, National Chung Hsing University, Taichung, Taiwan; 6 Tropical Medicine Research Center for Talaromycosis, Pham Ngoc Thach University of Medicine, Ho Chi Minh City, Vietnam; 7 Division of Infectious Diseases and International Health, Duke University School of Medicine, Durham, North Carolina, United States of America; University of Maryland, Baltimore, UNITED STATES OF AMERICA

## Introduction

**Fig 1:**
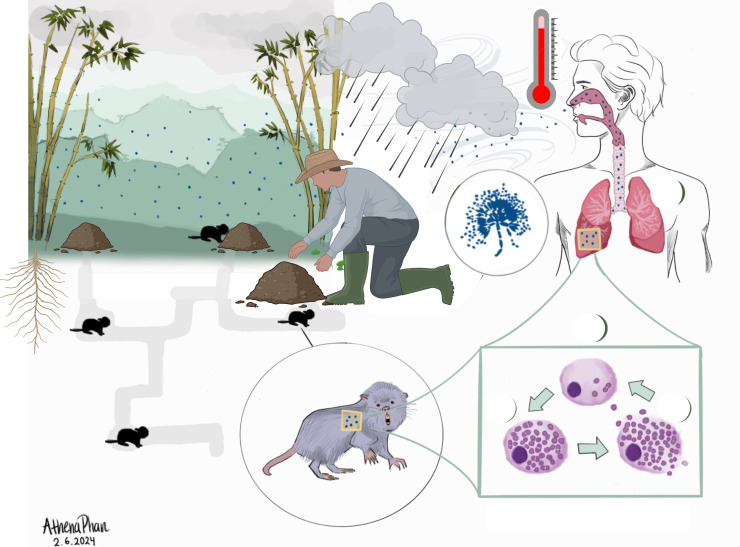
The environmental niches of *Talaromyces marneffei.* Original figure created by Athena Phan and adapted on BioRender by Lottie Brown (2025) (https://BioRender.com/2elo9zk). In the highlands of endemic regions, bamboo rats and their associated soil serve as a reservoir for *T. marneffei* growth. High temperatures, humidity, and precipitation during the tropical monsoon season provide favorable conditions for *T. marneffei.* Activities which disturb the soil, including agriculture and extreme wind and rainfall during the monsoon, are believed to facilitate aerosolization and dispersion of *T. marneffei* conidia and drive the acquisition of talaromycosis.

The dimorphic fungus *Talaromyces marneffei* causes talaromycosis, a life-threatening fungal disease and leading opportunistic infection in Southeast Asia. Talaromycosis is acquired through inhalation of *T. marneffei* conidia from the environment, and can develop as an acute infection or reactivation of a latent infection up to 50 years after initial exposure [1]. While the bamboo rat is the only known zoonotic reservoir for disease, there is no evidence of direct bamboo rat-to-human transmission. Rather, evidence suggests that *T. marneffei* exists in the soil associated with bamboo rats, which may be enriched with animal excreta and decaying tropical plants [2,3]. Activities which stir up the soil, including agriculture and extreme weather conditions during the tropical monsoon season, are believed to cause aerosolization of *T. marneffei* conidia and drive the acquisition of talaromycosis [3] (Fig 1). Hotspots for talaromycosis have been identified in the highlands of Southeast Asia, and residence or travel to these regions is a significant risk factor for developing disease. The clinical features of talaromycosis vary according to the immune status of the host. Disseminated, multisystem infection occurs among people with advanced HIV disease, while localized infections of the respiratory, gastrointestinal, or osteoarticular systems are more commonly seen in people without HIV [4]. Despite the significant disease burden (estimated >25,000 cases per year) and high mortality rate (15%–30% despite treatment), talaromycosis is a neglected disease, and research on *T. marneffei* has lagged behind other fungal pathogens [5]. In this *Pearls* review, we summarize the current understanding of talaromycosis pathogenesis and highlight key research gaps.

## Take a deep breath: The aerosolized transmission of *Talaromyces marneffei*

**Fig 2:**
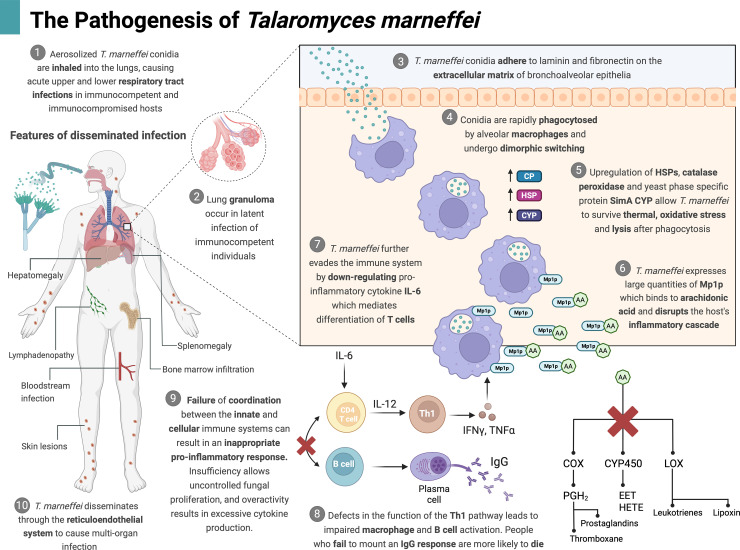
The pathogenesis of *Talaromyces marneffei.* Figure created in BioRender. Brown, L. (2025) (https://BioRender.com/2elo9zk). Key points (1) to (10) provide a summary of the pathogenesis of *T. marneffei.* Abbreviations: AA, arachidonic acid; CP, catalase peroxidase; COX, cyclooxygenase; CYP, cytochrome P; EET, epoxyeicosatrienoic acid; HETE, hydroxyeicosatetraenoic acid; HSP, heat shock proteins; IFN-γ, interferon gamma; IgG, immunoglobulin G; IL-6, interleukin-6; IL-12, interleukin-12; LOX, lipoxygenase; PGH_2_, prostaglandin H2; TNF-α, tumor necrosis factor-alpha.

Inhalation of aerosolized spores and hyphal elements from disturbed soil is considered the primary mode of transmission in humans, though rare cases of accidental needle inoculation and donor-derived infections have been reported (Fig 2) [1]. The respiratory tract is the portal of entry for talaromycosis, manifesting in the upper respiratory tract as oropharyngeal ulceration and obstructing tracheal masses, and in the lungs as pulmonary nodules, masses, and cavity lesions, which are increasingly reported among immunocompetent people with obstructive or cavitary lung disease [6]. In wild bamboo rats, the largest burden of *T. marneffei* has been recovered in the lungs (83%), followed by the liver (33%) and spleen (33%) [7]. A recent study demonstrated minimal innate cytokine response against *T. marneffei* in human bronchial epithelial cells as compared to human peripheral blood-derived macrophages, suggesting the potential role of bronchial epithelial cells as reservoirs for *T. marneffei* to evade immunosurveillance by phagocytes, from which the fungus lies dormant and reactivates when the host immunity is undermined [8]. These clinical and field observations, coupled with murine inhalation models demonstrating granulomatous pneumonia and disseminated infection to the liver and spleen, support the lungs being the primary portal of entry, and the establishment of acute, chronic, latent, and disseminated infection [6,9].

## Yeast mode is beast mode: Thermal dimorphism as a virulence factor for *Talaromyces marneffei*

*T. marneffei* is unique as the only one among over 100 *Talaromyces* species that exhibits thermal dimorphism, and among the few which are pathogenic to humans. When exposed to higher temperatures (32 °C to 40 °C), the saprophytic mold morphs into a pathogenic yeast [10]. Experimental evidence using human THP-1 macrophage cells indicates that thermal shapeshifting occurs rapidly inside the macrophage, within 12 to 24 hours [11,12]. Dimorphic switching is bidirectional and reproducible in the laboratory, although *in vitro* produced yeast cells differ morphologically to *ex vivo* and *in vivo* cells [13]. Morphological differences occur as a result of exposure to oxidative stress, changes in CO_2_ tension, acidic pH, high salinity, nutrient limitations, and host immune interactions inside the macrophage. These conditions activate the fungal stress response pathways that drive and maintain yeast-phase growth more robustly than *ex vivo* conditions [14]. Loss-of-function studies have highlighted key genes regulating the yeast phase transition. These include *abaA*—involved in asexual reproduction and yeast growth of dimorphic fungi; hybrid histidine kinase *slnA* and *drkA*—influencing germination inside the macrophage; and *msgA*—required for proper cell separation, among many others [10]. Dimorphism appears to be a key weapon in *T. marneffei’*s armamentarium and is associated with alteration of cell wall composition, and expression of yeast-phase specific cytochrome P450 (CYP) encoded by *simA*—which allows *T. marneffei* to survive lysis and degradation after phagocytosis through detoxification of reactive oxygen and nitrogen species and synthesis of protective secondary metabolites [15].

## Eaten only to prevail: *Talaromyces marneffei* survives engulfment by macrophages, hijacking the innate immune response

Following inhalation, *T. marneffei* conidia infiltrate through the bronchoalveolar epithelia by sticking to laminin and fibronectin proteins of the extracellular matrix via sialic acid, and to host glycosaminoglycans such as heparin and chondroitin sulphate B [16]. Inside the lungs, conidia are engulfed by alveolar macrophages via an L-arginine-dependent nitric oxide pathway and undergo dimorphic switching to unicellular yeasts, which then multiply by binary fission [17]. Thermal tolerance is a prerequisite for survival within the host. *T. marneffei* upregulates Heat Shock Proteins, which act as a shield against cellular damage and help maintain normal cellular function at mammalian body temperature [10]. In the fight against macrophage-induced oxidative stress, *T. marneffei* produces neutralizing enzymes such as catalase peroxidase encoded by *cpeA,* superoxide dismutase encoded by *sodA*, and a collection of aspartyl proteases encoded by the *pepA* and *pop* genes [10,18]. Like several other pathogenic fungi, melanin can be synthesized by *T. marneffei* and incorporated into the innermost layer of the yeast cell wall, which is advantageous for survival [11,19]. *In vitro* experiments have demonstrated that melanized yeast cells are more resistant to phagocytosis by macrophages and intracellular digestion by hydrolytic enzymes [20].

Transcription factors, AcuM and AcuK, facilitate survival under nutrient-scarce conditions within the macrophage, by enabling *T. marneffei* to exploit alternative carbon sources, and steal iron from the host cells [21]. AcuM, AcuK, and other members of the Acu gene family play additional roles in the oxidative stress response, by regulating the expression of antioxidant enzymes and alternative respiration enzymes [22]. *T. marneffei* further dodges the innate immune system by downregulating interleukin-6 (IL-6), a pro-inflammatory cytokine produced by bronchial epithelial cells, which mediates the differentiation of Th17 cells. *T. marneffei* plays its final trump card by secreting large quantities of Mp1p—a galactomannan protein encoded by *MP1*—which binds to arachidonic acid and disrupts the host proinflammatory cascade [23,24]. Through these cunning mechanisms, *T. marneffei* hijacks the macrophages as its personal incubators, growing and multiplying inside until they “pop”, releasing a flood of yeast progeny that spreads throughout the reticuloendothelial system.

## Peering through the host immune window, the journey from innate to adaptive immune responses

In addition to phagocytosis, the innate immune cells track and trace fungal wall components using sensors called pattern recognition receptors, including toll-like receptors (TLRs), and C-type lectin receptors. In a study of people with advanced HIV disease, those with genetic mutations in TLR genes (single-nucleotide polymorphisms or “SNPs”) were at an increased risk of severe disease and death [25]. Phagocytes of the innate immune system play a crucial role in disease control by secreting proinflammatory “fire-fighter” cytokines, including IL-12 and tumor necrosis factor-A (TNF-α) [26]. These cytokines drive the differentiation of T cells in the Th1 pathway, leading to the production of interferon gamma (IFN-γ), which enhances the activity of phagocytes in a positive feedback loop. In people with T cell deficiency, a breakdown in communication between the innate and cellular immune coordinating centers results in an inappropriate proinflammatory response, marked by a cytokine storm and uncontrolled release of TNF-α, IL-6, and IL-1β [27]. Enter the Casadevall’s damage response framework, which posits that immune insufficiency (i.e., due to advanced HIV disease) leads to unchecked fungal proliferation and dissemination, while immune overactivity (i.e., due to immune reconstitution inflammatory syndrome) results in uncontrolled inflammation, tissue damage, and poor prognosis [26–28].

## Immunity blunders give further clues into the host adaptive cellular response to *Talaromyces marneffei*

As with most invasive mycoses, the progression and severity of talaromycosis are highly dependent on the function of CD4 T cells and the Th1 response, hence the high disease burden among people with advanced HIV disease [29]. Development of talaromycosis among people with primary immunodeficiencies provides revelations into mechanisms of immune protection against *T. marneffei.* Anti-interferon-γ autoantibody (AIGA) syndrome—an adult-onset immunodeficiency common in individuals of Southeast Asian descent—is the most common underlying risk factor for talaromycosis among people without HIV, classically presenting with relapsing talaromycosis [30]. AIGAs block signal transducer and activator of transcription 1 (STAT1) phosphorylation and IL-12 production, sabotaging the Th1 response [31]. Defects in the Th1 pathway (e.g., mutations in CD40LG, STAT1, STAT3, IL2RG, IFNGR1, IL12RB1, CARD9, COPA, NFKB2) impair the activation of key proinflammatory cytokines, IFN-γ, and TNF-α, which are responsible for *T. marneffei* clearance [26,32].

## Much ado about nothing or under-appreciated? The adaptive humoral response to *Talaromyces marneffei*

The humoral immune system contributes to control of talaromycosis by producing *T. marneffei*-specific antibodies, which mark the fungus for destruction by opsonization, call for back up through complement activation, and neutralize the threat. There is recent clinical evidence for the importance of the humoral response in *T. marneffei* control. In a clinical trial cohort of 315 people with advanced HIV disease, one-third of patients who were able to rally a *T. marneffei-*specific immunoglobulin G (IgG) antibody response faired substantially better than those who were not, with significantly lower fungal burdens, less severe disease, and lower mortality, despite receiving the same antifungal treatment [33]. It is possible that this protective effect is a result of prior exposure to *T. marneffei* (or latent infection), which primes memory T cells of these individuals to mount an IgG response that is more effective at clearing *T. marneffei* [33]. This study sheds new light on the often underappreciated role of host humoral immune response to fungal infections and may have broad implications for adjunctive antibody therapy and antifungal vaccines.

## Mind the gaps: Priorities for future research

Numerous research gaps remain. Firstly, further work is needed to decode the innate and adaptive immune responses to *T. marneffei*. It is important to determine if *T. marneffei-*specific T cell responses underlie B cell-mediated immunity that drives disease severity and mortality independent of antifungal treatment [33]. Uncovering the causal relationship between T and B cell activation and which specific B and T cell epitopes are involved in immune protection could inform adjunctive immune therapeutic and vaccine development to prevent severe talaromycosis and death. Secondly, melanin—the pathogenic pigment—contributes to virulence and antifungal resistance in several fungi, and is produced by *T. marneffei* during infection, but its specific role in disease pathogenesis and severity, and its potential as a diagnostic and therapeutic target, is largely uncharted. Thirdly, genomic studies have revealed that *T. marneffei* possesses all the necessary genes for sexual reproduction, which raises suspicion for a cryptic sexual cycle, possibly in the bamboo rat reservoir or human host [34]. This could have clinical implications, as recombination can contribute to the emergence of hypervirulent and drug-resistant strains, as seen in other pathogenic fungi [35]. Further investigation is required to confirm the existence of a secretive sexual cycle and determine if it has any clinical relevance. Fourthly, there is a need for optimal *in vivo* models to study talaromycosis pathogenesis and therapeutics [36]. Most murine infection models use intravenous injection into the tail, which bypasses the natural route of respiratory infection and host innate immune response, thereby undermines the understanding of immune pathogenesis [36,37]. A murine inhalation model has been developed but presents a significant biohazard and requires high-level biosafety facilities, which limit its application [9]. Safer alternatives, such as oropharyngeal aspiration or intranasal instillation models, warrant further investigation. Identification of more invertebrate models could facilitate further studies of the complex interaction between pathogen and host, while also offering a cost-effective and scalable approach to evaluate novel therapeutics and vaccine candidates [38]. Finally, genomic studies have provided insights into the molecular basis of pathogenicity and revealed distinct geographical clades associated with varying levels of disease severity [39]. Further genetic and genomic studies will deepen our understanding of *T. marneffei* and its pathogenesis, as well as inform our understanding of its geographical variation and transmission dynamics. These will be important to identify potential diagnostics and therapeutic targets.
